# Anti-Inflammatory Activity and Composition of *Senecio salignus* Kunth

**DOI:** 10.1155/2013/814693

**Published:** 2013-04-14

**Authors:** Cuauhtemoc Pérez González, Roberto Serrano Vega, Marco González-Chávez, Miguel Angel Zavala Sánchez, Salud Pérez Gutiérrez

**Affiliations:** ^1^Universidad Autónoma Metropolitana-Xochimilco, AP 23-181, Mexico; ^2^Facultad de Ciencias Químicas, Universidad Autónoma de San Luis Potosí, Avenida Dr. Manuel Nava 6, Zona Universitaria, 78210 San Luis Potosí, SLP, Mexico

## Abstract

We investigated the anti-inflammatory activity of *Senecio salignus*. This medicinal plant is often used in Mexico for the treatment of fever and rheumatism. Chloroform and methanol extracts of the plant were tested on 12-O-tetradecanoylphorbol-13-acetate- (TPA-) induced edema in mice ears. The methanol extract of the plant inhibited edema by 36 ± 4.4% compared with the control, while the chloroform extract exhibited an even greater level of inhibition (64.1%). The chloroform extract was then fractionated, and the composition of the active fraction was determined by GC-MS. The anti-inflammatory activity of this fraction was then tested on TPA-induced ear edema in mice, and we found that the active fraction could inhibit edema by 46.9%. The anti-inflammatory effect of the fraction was also tested on carrageenan-induced paw edema in rats at doses of 100 mg/kg; a 58.9 ± 2.8% reduction of the edema was observed 4 h after administration of carrageenan, and the effect was maintained for 5 h.

## 1. Introduction 

Inflammatory diseases are a major cause of morbidity and mortality in the world. These diseases are mainly treated with nonsteroidal anti-inflammatory drugs (NSAIDs) and steroidal drugs, which have proven effective but can have negative side effects. For instance, NSAIDs may induce gastric and intestinal ulcers, anaemia, platelet inhibition in uterine motility, and, in some reported cases, an increased risk of myocardial infarction [1]. Steroidal anti-inflammatory drugs prevent or suppress inflammation but do not attack the root cause of the disease, and the prolonged use of these compounds can inhibit the synthesis of the inducible isoform of nitric oxide synthase enzyme and cause pituitary-adrenal suppression, hyperglycaemia, glycosuria, and an increased susceptibility to infections and peptic ulcers [2]. Therefore, searching for new molecules with anti-inflammatory activity but with fewer side effects is vital, and plants may represent a potential source of such compounds.

Ancient Mexican culture is rich in information on the plants used in traditional medicine, one of which is *Senecio salignus*. This plant is used to treat intermittent fevers and rheumatism, and in the state of Chiapas, it is used as an insecticide in corn stores and also as an ornamental plant [3, 4].

*S. salignus* is a leafy shrub with many branches; its leaves are sessile and are generally very narrow (up to 1.5 cm wide) and pointed, with numerous dense inflorescences and cones. Each inflorescence bears 5 to 6 bright yellow flowers. The plants grow in areas of desert scrub at altitudes less than 2870 m [5].

From the aerial parts of *S. salignus*, some pyrrolizidine alkaloids, lactones, furoeremophilanes, sesquiterpenes, and other compounds have been isolated [6]. However, there are no reports of anti-inflammatory activity for this plant.

In the present study, we investigated the anti-inflammatory activity of *S. salignus* and the composition of the active fraction of the plant. 

## 2. Materials and Methods

### 2.1. Biological Material

*Senecio salignus* Kunth (Compositae) was collected around Tenancingo in the state of Mexico in July 2010. Dr. Abigail Aguilar Contreras authenticated the species, and a voucher was deposited in the herbarium of the Instituto Mexicano del Seguro Social (IMSSM 15,546). The aerial parts of the plant were dried in the shade at room temperature.

### 2.2. Experimental Animals

The present study used male Wistar rats (180 to 200 g) and CD-1 male mice (20 to 25 g) provided by the Unidad de Producción y Experimentación de Animales de Laboratorio (UPEAL) at the Universidad Autónoma Metropolitana Xochimilco. The animals were provided with food and water *ad libitum* and housed in a facility with light and dark periods of 12 hours.

All experiments were carried out according to the guidelines of laboratory animal care of the Guide for the Care and Use of Laboratory Animals [7].

### 2.3. Extract Preparation

A mixture of 500 g of dried, ground leaves and 3.5 L of chloroform or methanol was placed into a 5 L flask with a reflux condenser. The mixture was heated for 4 h at boiling temperature and then cooled and filtered; the solvent was then evaporated in a rotary evaporator to dryness under reduced pressure. The yield was 0.6% and 1.2%, respectively.

### 2.4. Chloroform Extract Fractionation

The chloroform extract was separated by column chromatography; the column was packed with silica gel (Kieselgel 60, 70–230 mesh ASTM), which was prepared using hexane as the mobile phase, and then the polarity was increased with ethyl acetate. Fractions of 100 mL were collected and compared by thin-layer chromatography; fractions with the same chromatographic pattern were then pooled. The resulting fractions were tested on ear edema in mice induced by TPA, and the composition of the fractions with the highest activity was then determined.

### 2.5. Active Fraction Analysis (AF)

The analysis was performed on a gas chromatograph coupled to a mass spectrometer (Agilent Technology, model 6890N); this was coupled to a mass selective detector (model 5973) with a DB-5HT capillary column (15 m in length, 0.25 mm internal diameter, and 0.10 *μ*m film thickness). We used a temperature program starting at 100°C for 3 min with a heating rate of 10°C per min up to 320°C; this temperature was maintained for 5 min. The splitless injection was performed at a ratio of 1 : 100, and the injector temperature was 320°C. The spectra were determined at 70 eV, and the mass range analysed was from 33 to 800 m/z. The compounds were identified from the mass spectra and by comparing the spectra to the spectra reported in the NIST database (Wiley09/NIST11).

### 2.6. Anti-Inflammatory Activity

#### 2.6.1. 12-O-Tetradecanoylphorbol-13-Acetate- (TPA-) Induced Mouse Ear Edema

The model for TPA-induced edema in mouse ears has been described previously [8]. A solution containing 2.5 *μ*g of TPA in 25 *μ*L of acetone was applied topically to the inner and outer surfaces of the right ears in a group of eight male CD1 mice. Thirty minutes later, 2.0 mg of the chloroform extract or AF or indomethacin dissolved in acetone was topically applied to the right ear, and acetone was applied to the left ear. Six hours later, the animals were sacrificed, and 6 mm plugs of the central portion of both ears were weighed. The percentage inhibition of edema was determined.

#### 2.6.2. Carrageenan-Induced Rat Paw Edema

The model for carrageenan-induced edema in the rat paw has been described previously [9]. Paw edema was induced by intradermal injection of 0.1 mL of a 1% carrageenan suspension in the left hind footpad. One hour prior to carrageenan injection, groups of eight rats each were treated with 50, 100, 200, or 400 mg/kg CESS or AF, while another group received 8 mg/kg indomethacin. The control group received the vehicle alone (polyvinyl pyrrolidone (PVP)). The paw volume was measured by the volume displacement method using a plethysmometer (Ugo Basile) at 1, 2, 3, 4, and 5 h after carrageenan administration, and the percentage of edema inhibition was determined [10].

#### 2.6.3. Acute Toxicity

AF was orally administered as a single dose at different concentrations (312–5000 mg/kg) to groups of mice (*n* = 5). After administration, the animals were observed under open-field conditions for a 72 h period. The number of animal deaths and signs of clinical toxicity were recorded [11].

### 2.7. Statistical Analysis

Data are expressed as the mean ± S.E.M. The statistical analysis was performed using Student's *t*-test (*P* < 0.05), and ANOVA followed by Dunnett's test (*P* < 0.05) was used to determine significance.

## 3. Results and Discussion

The methanol extract of *S. salignus* inhibited TPA-induced ear edema by 36.4 ± 4.4%, while the chloroform extract (CESS) diminished the ear edema by 64.1 ± 3.9%; the effect was higher than that obtained with indomethacin (41.5 ± 4.3%). The study was then continued with the CESS, which was separated by column chromatography to give 12 fractions. Fraction 5 (AF) (hexane: AcOEt 7 : 3) showed the highest inhibition of TPA-induced ear edema (46.9 ± 5.3%). 

The inflammation induced by TPA is mediated by protein kinase C, which stimulates phospholipase A2 [12] and cyclooxygenase, resulting in the release of arachidonic acid and prostaglandin E2 [13]. The AF displayed good activity, which suggested that at least one of the compounds present in *S. salignus* may inhibit the production of protein kinase C, resulting in the observed effect.

CESS and AF were also tested on carrageenan-induced paw edema in rats, and the results are shown in Tables 1 and 2.

The activity of CESS at doses of 50, 100, 200, and 400 mg/kg 3 h after carrageenan administration was similar to that presented with indomethacin. After 5 h, the activity of the extract diminished at doses of 50 and 100 mg/kg, whereas at 200 and 400 mg/kg (63.7 ± 5.6% and 55.5 ± 5.9%, resp.), the inhibition was similar to that by indomethacin (66.8 ± 3.6%).

In contrast, AF (Table 2) at a dose of 50 mg/kg inhibited the edema by only 38.9 ± 3.3% 5 h after carrageenan administration; however, at doses of 100, 200, and 400 mg/kg, the effect (60.1 ± 4.7, 62.9 ± 2.0, and 80.1 ± 4.5%, resp.) was similar to that obtained with indomethacin (62.2 ± 3.9%). The best activity was observed at doses of 400 mg/kg, but at this dose, AF has some toxic effects.

Leukocyte migration to injured tissue is an important aspect of the inflammatory process. The release of several mediators of the phlogistic response, including histamine and serotonin, is responsible for the immediate inflammation response [14], whereas kinins and prostaglandins mediate the prolonged response [15]. In contrast, some plant ingredients significantly inhibit the biosynthetic pathways of inflammation mediators [12]. AF showed an effect in the animal model, suggesting that its anti-inflammatory activity could inhibit the production of these mediators.

AF was analysed by GC-MS, and its composition is shown in Table 3. We found a total of 185 compounds, of which 50 were identified. In Table 3, only those compounds whose concentration was higher than 0.2% have been shown; the major compounds were hexadecanoic acid (3.76%), (Z, Z)-octadecadienoic acid (7.5%), (Z, Z, Z)-9,12,15-octadecatrienoic acid (5%), squalene (5.17%), and nonacosane (10.11%).

Nonacosane is also found in the essential oil of *Artemisia annua*, a plant whose anti-inflammatory activity on carrageenan-induced edema has been attributed to this compound [16].

Reports in the literature also indicate that (Z, Z, Z)-9,12,15-octadecatrienoic acid prevents inflammatory problems as a precursor of prostaglandins. Simopoulos [17, 18] found that this compound and (Z, Z)-9,12-octadecadienoic acid could be used in the treatment of health problems such as type 2 diabetes, some types of cancer, ulcerative colitis, psoriasis, and rheumatoid arthritis. 

These facts suggested that (Z, Z, Z)-9,12,15-octadecatrienoic acid, (Z, Z)-9,12-octadecadienoic acid, and nonacosane might be responsible for the observed anti-inflammatory activity.

AF produced a slight change in the normal colour of the kidney at doses of 625 mg/kg. However, at doses of 312.5 mg/kg, no damage was observed in any of the animals' organs. This dose is higher than the active dose of AF (100 mg/kg). These results represent the first step in the process of obtaining a standardised extract and developing a phytomedicine.

## 4. Conclusions

The active fraction of *S. salignus* was separated from the chloroform extract. The composition was characterised by GC-MS. AF exhibited anti-inflammatory activity in the inflammation models used in this work when administered topically and orally. 

## Figures and Tables

**Table 1:** Anti-inflammatory effect of the chloroform extract of *Senecio salignus* on rat paw edema induced by carrageenan.

Time	Doses				
	Indomethacin	Doses of *Senecio salignus *			
	8 mg/kg	50 mg/kg	100 mg/kg	200 mg/kg	400 mg/kg
1 h	37.1 ± 3.9*	31.2 ± 4.8	35.7 ± 7.3*	32.4 ± 4.1*	48.1 ± 2*
2 h	62 ± 4.1*	50.8 ± 5.7*	56.1 ± 5.2*	55.4 ± 4.7*	56.8 ± 7*
3 h	63 ± 4.2*	56.7 ± 6.7*	54 ± 4*	60.6 ± 4.9*	64.1 ± 4*
4 h	57.9 ± 3.5*	40.8 ± 4.3*	51.1 ± 5*	60.1 ± 2.3*	59.7 ± 6.2*
5 h	66.8 ± 3.6*	38.5 ± 8.8	45.3 ± 4.2*	63.7 ± 5.6*	55.5 ± 5.9*

Results are expressed as percentage of inhibition and mean of eight determinations ± SE. One-way ANOVA, Dunnett test *P < 0.05 (*P* = 0.04) for the comparison of *S. salignus* with indomethacin-treated groups.

**Table 2:** Anti-inflammatory effect of AF on paw edema induced by carrageenan.

Time (H)	% Inhibition				
	Indomethacin	*Senecio salignus *			
	8 mg/kg	50 mg/kg	100 mg/kg	200 mg/kg	400 mg/kg
1	15.1 ± 2.8*	10.3 ± 4.02*	15.03 ± 3.1*	17.5 ± 2.3*	27.5 ± 3.8
2	28.3 ± 3.8*	21.3 ± 2.4*	27.8 ± 3.1*	29.1 ± 3.1*	34.5 ± 2.2*
3	43.4 ± 3.2*	26.6 ± 2.1	47.5 ± 4.02*	44.6 ± 3.8*	57.3 ± 2.6
4	61.1 ± 3.8*	37.2 ± 4.6	58.9 ± 2.8*	63.5 ± 2.2*	70.3 ± 4.5
5	62.2 ± 3.9*	38.9 ± 3.3	60.1 ± 4.7*	62.9 ± 2.04*	80.1 ± 4.5

Results are expressed as percentage of inhibition and mean of eight determinations ± SE. One-way ANOVA, Dunnett test *P < 0.05 for comparison of *S. salignus* with indomethacin-treated groups.

**Table 3:** *Senecio salignus* composition of the active fraction.

Retention time (min)	Compound	% Relative
4.670	Isocaryophyllene	0.61
4.833	*α*-cadinene	1.0
7.524	Tetradecanoic acid	0.57
9.701	Hexadecanoic acid	3.76
10.163	3-(6-Methoxy-3-methyl-2-benzofuranyl) ethyl butyrate	0.41
11.286	(Z, Z)-9.12-Octadecadienoic acid	7.5
11.337	(Z, Z, Z)-9,12,15-Octadecatrienoic	5.0
11.569	Octadecanoic acid	1.24
13.283	Eicosanoic acid	1.53
14.860	Docosanoic acid	0.88
15.597	Tricosanoic acid	0.24
16.111	Heptacosane	0.84
16.325	Tetracosanoic acid	0.54
16.822	Octacosanol	0.46
16.976	Squalene	5.17
17.576	Nonacosane	10.11
17.713	Hexacosanoic acid	0.63
18.151	Triacontane	1.45
18.990	Octacosanoic acid	0.53
19.393	Dotriacontane	0.32
19.993	Tritriacontane	1.15
20.199	Triacontanoic acid	0.56
20.807	Acetate(3*α*-21*α*)-*α*-neogammacer-22(29)-en-3-ol	1.56
21.313	Dotriacontanoic acid	0.45
